# Stingless Bee Larvae Require Fungal Steroid to Pupate

**DOI:** 10.1038/s41598-018-19583-9

**Published:** 2018-01-18

**Authors:** Camila R. Paludo, Cristiano Menezes, Eduardo A. Silva-Junior, Ayrton Vollet-Neto, Andres Andrade-Dominguez, Gleb Pishchany, Lily Khadempour, Fabio S. do Nascimento, Cameron R. Currie, Roberto Kolter, Jon Clardy, Mônica T. Pupo

**Affiliations:** 1 School of Pharmaceutical Sciences of Ribeirão Preto, University of São Paulo, Ribeirão Preto, São Paulo 14040-903 Brazil; 2 Brazilian Agricultural Research Corporation, Embrapa Amazônia Oriental, Belém, 66095-100 Brazil; 3 Department of Biology, FFCLRP, University of São Paulo, Ribeirão Preto, São Paulo 14040-901 Brazil; 4 Department of Microbiology and Immunobiology, Harvard Medical School, Boston, Massachusetts, MA 02115 USA; 5 Department of Bacteriology, University of Wisconsin, Madison, Wisconsin, WI 53706 USA; 6 Department of Zoology, University of Wisconsin, Madison, Wisconsin, WI 53706 USA; 7 Department of Biological Chemistry and Molecular Pharmacology, Harvard Medical School, Boston, Massachusetts, MA 02115 USA

## Abstract

The larval stage of the stingless bee *Scaptotrigona depilis* must consume a specific brood cell fungus in order to continue development. Here we show that this fungus is a member of the genus *Zygosaccharomyces* and provides essential steroid precursors to the developing bee. Insect pupation requires ecdysteroid hormones, and as insects cannot synthesize sterols *de novo*, they must obtain steroids in their diet. Larval *in vitro* culturing assays demonstrated that consuming ergosterol recapitulates the developmental effects on *S. depilis* as ingestion of *Zygosaccharomyces* sp. cells. Thus, we determined the molecular underpinning of this intimate mutualistic symbiosis. Phylogenetic analyses showed that similar cases of bee-*Zygosaccharomyce*s symbiosis may exist. This unprecedented case of bee-fungus symbiosis driven by steroid requirement brings new perspectives regarding pollinator-microbiota interaction and preservation.

## Introduction

Bees originated in the wasp family Crabronidae^1^ during the Cretaceous (113–132 million years ago) when angiosperms became the dominant flowering plants on the planet, and bee ancestors became phytophagous^2^. This switch from predation to phytophagy led to the remarkable diversification of bees^1,2^, which today include some 25,000 described species^3^. Recently, it was described that a Brazilian bee, besides pollen and honey, also needs to consume fungal cells to survive^4^. The stingless bee *Scaptotrigona depilis* (Hymenoptera: Apidae: Meliponini) has a fascinating life history, requiring the consumption of a specific brood fungus during larval stage. After *S. depilis* eggs hatch, a white microbial growth becomes visible at the boundary of the brood cell wall and the surface of the larval food supply^4^. When first described in 1974, the white microbial growth was hypothesized to be a pathogenic microorganism^5^. However, it was demonstrated that the microbial mass is composed largely of a symbiotic fungus, initially identified as *Monascus* sp., which is eaten by the larvae that require it to complete development^4^. The presence of similar fungus-growing phenomenon was also observed inside brood cells from other stingless bees, such as *Tetragona clavipes* and *Melipona flavolineata*^6^.

The associations between insects and microbes are mediated by several mechanisms, such as nutritional supply, chemical defense and communication. Symbiont microbes are known to produce a range of defensive compounds to protect their hosts against predators, parasites and pathogenic microorganisms^7^. In most cases, the active compound produced by the defensive microbiota is selective against the system-associated parasite^7,8^. Attine ants, for example, cultivate fungi for food^9,10^. The fungal gardens are protected by actinobacteria, which produce small molecules to inhibit the growth of parasitic fungi in the genus *Escovopsis*^11,12^. Symbiotic bacteria can also produce pheromones used by their hosts. In the case of locusts *Schistocerca gregaria*, an aggregation pheromone is produced by gut-associated bacteria^13^. These examples show how complex and fascinating are such associations.

The discovery of the beneficial symbiosis between *S. depilis* and the brood cell fungus initiated an important change in our ongoing bee microbiota studies. We sought the molecular basis of this symbiosis. Insects metamorphosis involves the participation of ecdysteroids, which are essential sterol-derived molting hormones that induce the major transformations from immature individuals to adults^14,15^. Since insects cannot biosynthesize sterols *de novo*, ecdysteroids are produced from dietary sterols^15^. Here, we show that *Zygosaccharomyces* sp. is the fungus eaten by *S. depilis* larvae and that fungus consumption provides ergosterol to developing bees, allowing successful pupation. Importantly, phylogenetic analyses showed that other cases of bee-*Zygosaccharomyces* association may exist, opening new perspectives regarding bee-microbe symbiosis.

## Results

### *Zygosaccharomyces* sp. is the fungus eaten by *S. depilis* larvae

**Figure 1:**
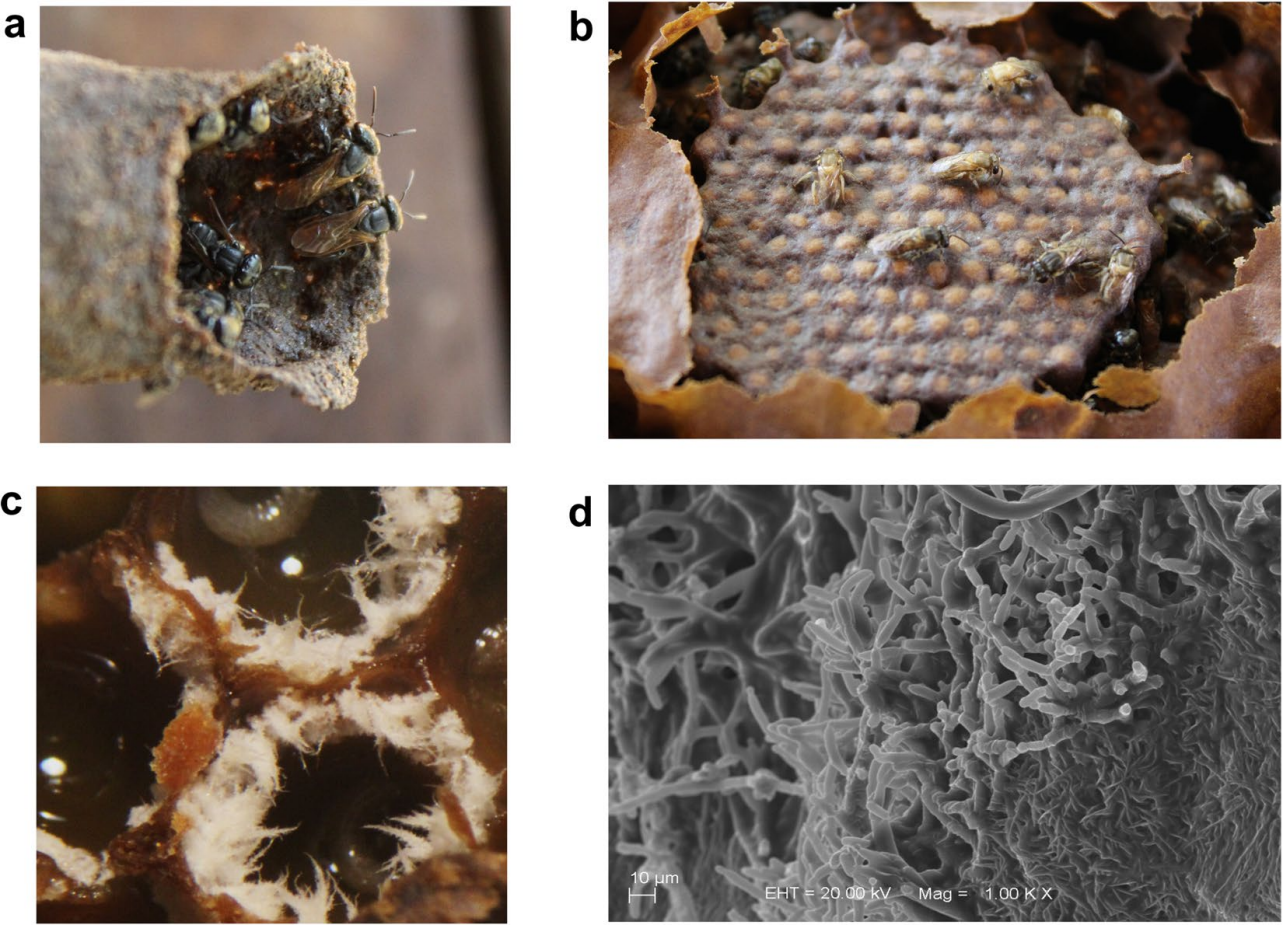
Characteristics of *S. depilis* and its food-fungus. (**a**) *S. depilis* in the entrance of the colony. (**b**) Brood cells with newly emerged bees. (**c**) *Zygosaccharomyces* sp. pseudomycelium inside *S. depilis* brood cells. (**d**) Scanning electron microscopy of *S. depilis* brood cell fungus.

**Figure 2:**
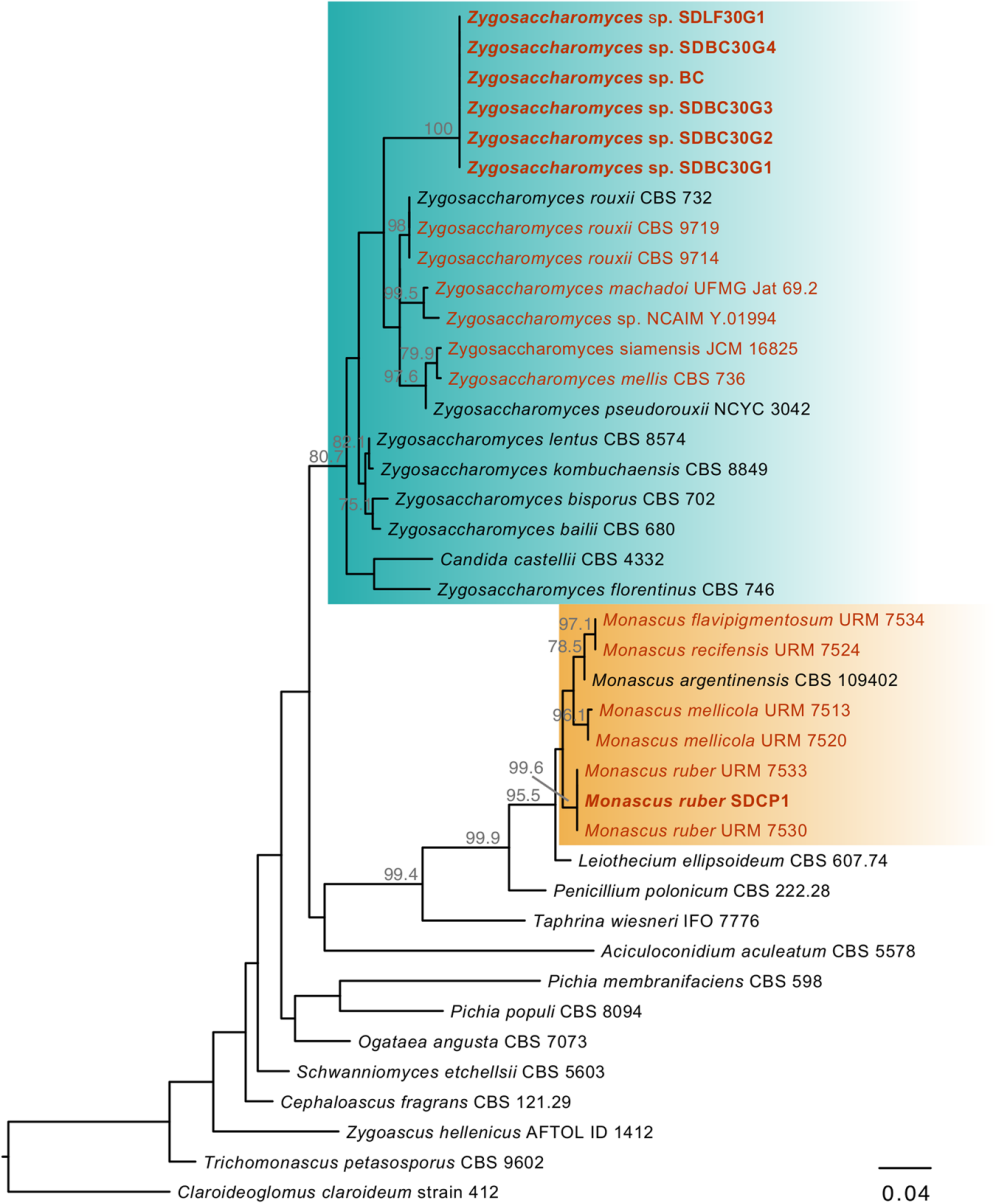
Phylogeny of the LSU gene regions showing *Zygosaccharomyces* spp. clade in the blue box and *Monascus* spp. clade in the orange box. Bee-associated strains were highlighted in red, and *S. depilis*-associated microorganisms were also highlighted in bold.

In an effort to further understand the *S. depilis* (Fig. 1a) larvae-fungus interaction, the microbial mass accumulating inside *S. depilis* brood cells (Fig. 1b,c), and which the larvae eat, was carefully collected and plated on PDA and ISP-2 agar. However, no growth on these plates was obtained. Both PDA and ISP-2 are relatively low osmolarity media. Since the brood cells are filled with very high osmolarity larval food supply, we decided to test for microbial growth in very high osmolarity medium. Indeed, when we used a medium with 30% glucose a yeast from the genus *Zygosaccharomyces* grew. This fungus grows from the cerumen (Fig. 1c,d) and can be isolated from larval food 3–4 days after the eggs have been laid. Genus-level identification was determined through 18 S and 26 S rRNA DNA sequencing and molecular phylogenetics on isolated strains (Fig. 2, Table 1).

**Table 1:** Strains and sequences used in the phylogeny.

Species name	Strain number	LSU Accession number	Isolation source
*Aciculoconidium aculeatum*	CBS 5578	KY106090	*Drosophila occidentalis*
*Candida castellii*	CBS 4332	KY106389	Soil
*Cephaloascus fragrans*	CBS 121.29	U40091	Unknown
*Claroideoglomus claroideum*	Isolate 412	DQ469099	Temperate agricultural grassland
*Leiothecium ellipsoideum*	CBS 607.74	FJ358285	Soil, between rocks
*Monascus argentinensis*	CBS 109402	KY645974	Soil sample
*Monascus flavipigmentosum*	URM 7534	KY511780	Pollen of *Melipona scutellaris*
*Monascus mellicola*	URM 7513	KY511759	Honey of *Melipona scutellaris*
*Monascus mellicola*	URM 7520	KY511766	Pollen
*Monascus recifensis*	URM 7524	KY511770	Pollen of *Melipona scutellaris*
*Monascus ruber*	SDCP1	MF196245	*Scaptotrigona depilis* brood cells cerumen
*Monascus ruber*	URM 7530	KY511776	Inside nest of *Melipona scutellaris*
*Monascus ruber*	URM 7533	KY511779	Inside nest of *Melipona scutellaris*
*Ogataea angusta*	CBS 7073	KY108669	*Drosophila pseudobscura*
*Penicillium polonicum*	CBS 222.28	JN939272	Soil
*Pichia membranifaciens*	CBS 598	KY108894	Beer
*Pichia populi*	CBS 8094	U75427	*Populus trichocarpa*
*Schwanniomyces etchellsii*	CBS 5603	KY109607	Human faeces
*Taphrina wiesneri*	IFO 7776	AY548292	Unknown
*Trichomonascus petasosporus*	CBS 9602	KY109917	Unknown
*Zygoascus hellenicus*	AFTOL ID 1412	FJ176885	Unknown
*Zygosaccharomyces bailii*	CBS 680	U72161	Unknown
*Zygosaccharomyces bisporus*	CBS 702	U72162	Unknown
*Zygosaccharomyces florentinus*	CBS 746	U72165	Sulphited grape must
*Zygosaccharomyces kombuchaensis*	CBS 8849	AF339904	Kombucha tea
*Zygosaccharomyces lentus*	CBS 8574	AF339888	Orange juice
*Zygosaccharomyces machadoi*	UFMG Jat 69.2	AF432228	*Tetragonisca angustula* refuse pile
*Zygosaccharomyces mellis*	CBS 736	U72164	Honey
*Zygosaccharomyces pseudorouxii*	NCYC 3042	AJ555406	Sugar
*Zygosaccharomyces rouxii*	CBS 9714	AJ716118	*Bombus pascuorum* digestive tract
*Zygosaccharomyces rouxii*	CBS 9719	AJ716119	*Bombus terrestris* honey
*Zygosaccharomyces rouxii*	CBS 732	U72163	Grape must
*Zygosaccharomyces siamensis*	JCM 16825	AB565756	*Apis mellifera* honey
*Zygosaccharomyces* sp.	SDBC30G4	MF194021	*Scaptotrigona depilis* brood cells
*Zygosaccharomyces* sp.	SDBC30G3	MF194019	*Scaptotrigona depilis* brood cells
*Zygosaccharomyces* sp.	SDBC30G2	MF194020	*Scaptotrigona depilis* brood cells
*Zygosaccharomyces* sp.	SDLF30G1	KY766952	*Scaptotrigona depilis* larval food
*Zygosaccharomyces* sp.	SDBC30G1	KY766262	*Scaptotrigona depilis* brood cells
*Zygosaccharomyces* sp.	BC	MF280267	DNA sample from *Scaptotrigona depilis* brood cells fungus
*Zygosaccharomyces* sp.	NCAIM Y.01994	JF830782	*Apis mellifera* honeycomb

The fungus *Monascus ruber*, described as the symbiotic microorganism in a previous investigation^4^, was only isolated from *S. depilis* cerumen. To examine whether this fungus was present in the fungal material eaten by the larvae, we collected the microbial mass growing in approximately 20 brood cells of *S. depilis*, pooled the material and extracted its DNA. The 18 S rRNA and 26 S genes were amplified and sequenced. The sequences from the fungus collected directly in brood cells were identical to those from *Zygosaccharomyces* sp. SDBC30G1 (Fig. 2, Supplementary Fig. 1a). To confirm the absence of *M. ruber* SDCP1 in the material eaten by larvae, specific primers for the 18 S gene regions of *Zygosaccharomyces* sp. SDBC30G1 and *M. ruber* SDCP1 were designed (Supplementary Table 1). Using these specific primers, the 18 S regions of each isolated fungus were amplified as controls and compared with the amplicon from the material collected inside brood cells of *S. depilis*. The results showed that *M. ruber* was not present, but *Zygosaccharomyces* sp. 18 S was amplified (Supplementary Fig. 1b). Taken together, these data confirmed that *M. ruber* can be found in the cerumen, but only *Zygosaccharomyces* sp. develops the aerial filaments inside brood cells that are eaten by *S. depilis* larvae.

### *Zygosaccharomyces* sp. has distinctive morphological characteristics

**Figure 3:**
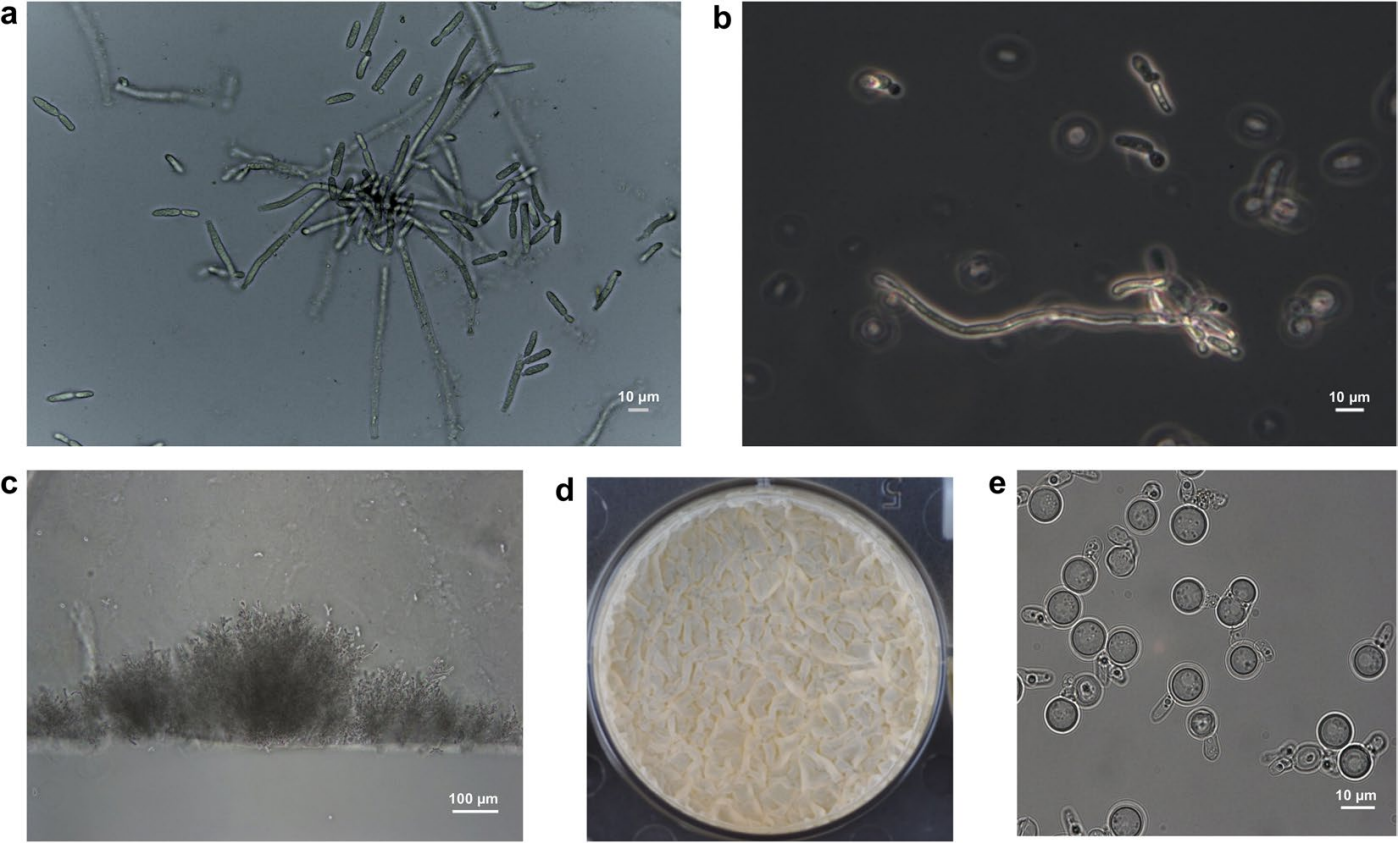
Growth characteristics of *S. depilis*-associated *Zygosaccharomyces* sp. (**a**) Pseudomycelium of *Zygosaccharomyce*s sp. in *S. depilis* brood cells. (**b**) Cells of *Zygosaccharomyce*s sp. SDBC30G1 cultured in 30G broth, pH 4.5. (**c**) Agar invasion of *Zygosaccharomyces* sp. SDBC30G1 colony (15GF agar medium, pH 4.5, 30 °C, after 10 days). (**d**) Pellicle formation of *Zygosaccharomyces* sp. SDBC30G1 (5 mL, 15GF broth, pH 4.5, static condition, 30 °C, inoculum OD_600_ 0.1, 6-well plates, after 7 days). (**e**) Ascospores of *Zygosaccharomyces* sp. SDBC30G1 (30% glucose and 0.5% of yeast extract liquid medium, static condition, 30 °C, inoculum OD_600_ 0.1, after 7 days).

**Figure 4:**
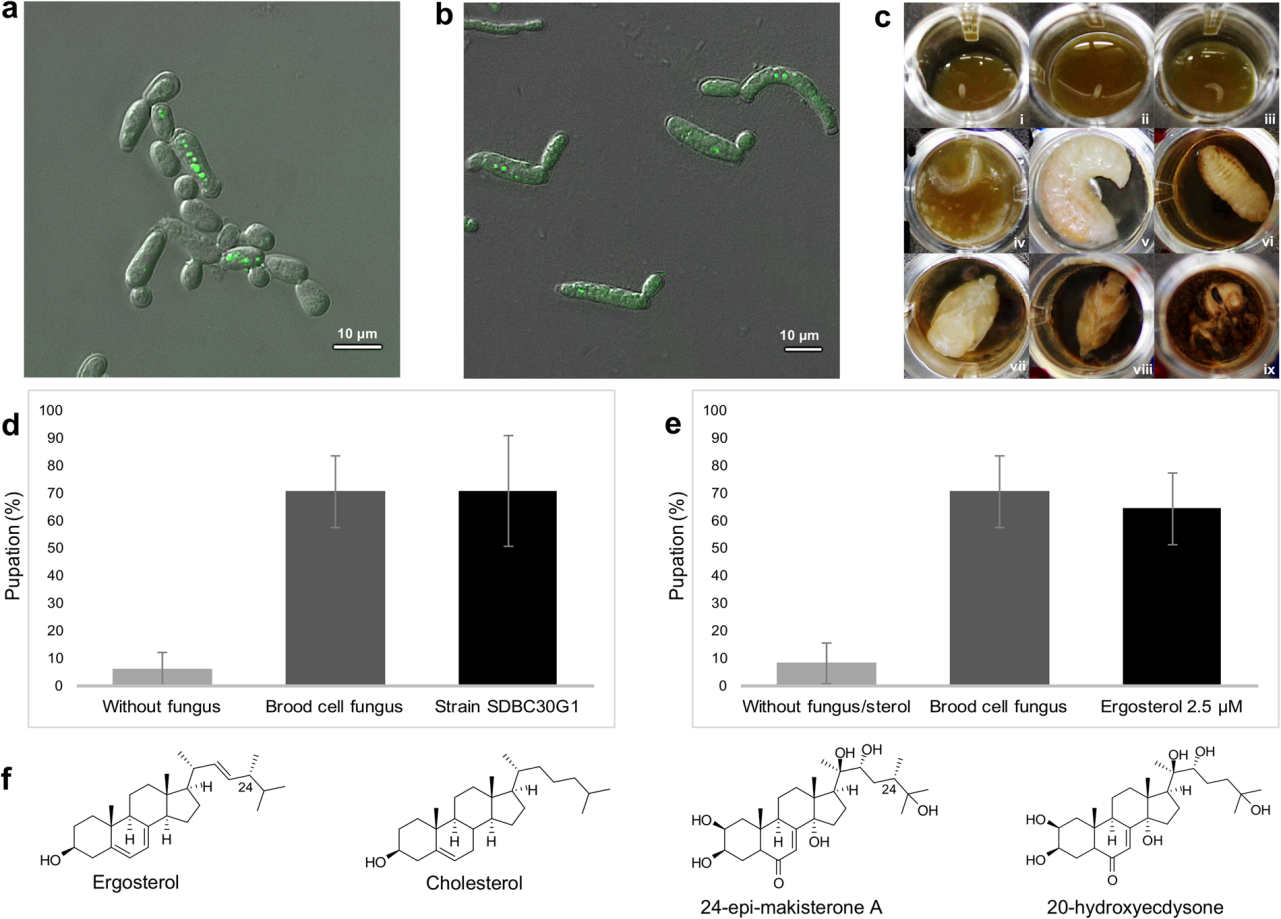
*Zygosaccharomyces* sp. lipid droplets and *S. depilis* pupation experiments. (**a**) Fluorescence microscopy of *Zygosaccharomyces* sp. fixed cells stained by Nile Red to show the presence of cytoplasmic lipid droplets under laboratory conditions and (**b**) natural conditions (cells of the fungus collected directly from brood cells of *S. depilis*). (**c**) Different stages of larval development: (i) 1–2 days-old egg, (ii) 3–4 day-old egg, (iii) 1 day-old larva, (iv) 3–4 day-old larvae (*Zygosaccharomyces* sp. growth observed), (v) 6–10 day-old larva, (vi) 15–18 day-old pre-pupa, (vii) 20–25 day-old pupa, (viii) 30–34 day-old pupa, (ix) 35–40 day-old emerging bee. (**d**) Percentage of larvae that completed metamorphosis *in vitro* without microorganism inoculation (first bar), with fungus collected directly from brood cells (second bar) and with isolated *Zygosaccharomyces* sp. SDBC30G1, cultivated in laboratory (third bar), in three different experiments (*N* = 48 per treatment, Cochran-Mantel-Haenszel, *P* < 0.0001 compared with uninoculated control). (**e**) Percentage of larvae that completed metamorphosis *in vitro* without fungal or sterol inoculation (first bar), with fungus collected directly from brood cells (second bar) and with ergosterol at 2.5 µM added in the larval food (third bar), in three different experiments (*N* = 48 per treatment, Cochran-Mantel-Haenszel, *P* < 0.0001 compared with untreated control). (**f**) Chemical structures of ergosterol, cholesterol, 24-epi-makisterone A and 20-hydroxyecdysone.

*Zygosaccharomyces* sp. displays unique growing characteristics in brood cells. This yeast exhibits pseudomycelium formation (Figs 1c and 3a), which has not been previously described for any other member of the *Zygosaccharomyces* genus^16^. Under laboratory conditions, *Zygosaccharomyces* sp. SDBC30G1 shows high degree of pseudohyphae formation, with some structures reaching 100 μm in length without septa (Fig. 3b). *Zygosaccharomyces* sp. SDBC30G1 invades agar (Fig. 3c) and forms floating pellicles (Supplementary Movie 1, Fig. 3d). Like other members of the *Zygosaccharomyces* genus^16^, strain SDBC30G1 forms spores during starvation (Fig. 3e). Additionally, we have observed cytoplasmic accumulation of lipid droplets (LDs) by *Zygosaccharomyces* sp. under laboratory conditions (Fig. 4a) and when it is growing in the brood cells (Fig. 4b). LDs accumulation suggested a nutritional value of the yeast to the larvae.

### *S. depilis*-associated *Zygosaccharomyces* sp. helps larval development

To verify whether the isolated *Zygosaccharomyces* sp. SDBC30G1 had the capacity to help larvae development, we performed *in vitro* larval development assays using 96-well plates with the isolated strain. To that end, larval cultures were initiated by transferring *S. depilis* eggs into 33 μL of larval food collected with a pipette from brood cells containing eggs. The experiment was performed using eggs to avoid contamination with *Zygosaccharomyces* sp. found in the larval food during larval stage. Transferring the eggs instead of larvae also avoided collecting larvae that had already eaten the resident fungus. Before the eggs hatched, no *Zygosaccharomyces* sp. could be grown from the larval food. Notwithstanding, a freeze-thawing sterilization method was used on the collected larval food to further reduce the chance of contaminants.

After the eggs hatched (3–4 days at 29 °C), we inoculated 16 larvae-containing wells with 1 μL of *Zygosaccharomyces* sp. SDBC30G1 (OD_600_ 45) or 1 μL of an inoculum prepared with the fungus collected directly from brood cells (OD_600_ 38). The inocula were prepared using a 30% glucose broth, and 1 μL of this sterile broth was used as a negative control. The experiment was performed three times using eggs and fungi from, at least, three different *S. depilis* colonies (Fig. 4c). The average percentage of larvae that completed metamorphosis with the fungus collected directly from brood cells and with the isolated *Zygosaccharomyces* sp. SDBC30G1 was the same (71%); while the frequency of morphogenesis in non-inoculated larvae was significantly lower (6%) (Cochran-Mantel-Haenszel, *P* < 0.0001) (Fig. 4d, Supplementary Fig. 2). Larvae that were not supplemented with fungus showed a delay in development when compared to larvae that ate natural and isolated *Zygosaccharomyces* sp. SDBC30G1 inoculum. The non-inoculated larvae were arrested in the larval stage, and after two or three weeks they died.

### Fungal steroid promotes larval metamorphosis

Based on the *in vitro* larval assays and *Zygosaccharomyces* sp. LDs accumulation, we hypothesized that this fungus could be a source of sterols for ecdysteroids and other sterol production. *Zygosaccharomyces* sp. sterols were extracted and analyzed by GC-MS, and ergosterol was the major one detected (Supplementary Fig. 3). To investigate the effect of ergosterol on *S. depilis* larval development, *in vitro* larval culturing experiments were performed where, instead of *Zygosaccharomyces* sp. SDBC30G1, we added pure ergosterol. A positive control was performed using *Zygosaccharomyces* sp. collected directly from brood cells.

The average percentage of pupation after consumption of ergosterol at 0.25 µM was 38% and with ergosterol at 2.5 µM was 65%. Larvae that ate fungal cells pupated at 71% and larvae reared without fungus or sterol displayed a pupation percentage of 8% (Cochran-Mantel-Haenszel, *P* < 0.0001; Fig. 4e, Supplementary Fig. 4). The morphogenesis rates were statistically similar when larvae were reared with the *Zygosaccharomyces* sp. cells or ergosterol at 2.5 µM (Cochran-Mantel-Haenszel, *P* > 0.05), indicating that pure ergosterol supports larval development.

### *S. depilis* pupae chemical profile

To investigate the ecdysteroids present in *S. depilis* pupae in natural condition, we collected *S. depilis* pupae from three different colonies, extracted them separately with methanol and the resulting extracts were analyzed using high-resolution LC-MS. The ion corresponding to makisterone A (MaA) or epi-makisterone A (epi-MaA) was detected in all extracts (Supplementary Fig. 5). The HRMS, *m/z* 495.3314 [M + H]^+^ (C_28_H_47_O_7_, error 0.5 ppm), and the MS/MS profile are in accordance with literature data for the isomers MaA or epi-MaA^15^. The ecdysteroids ecdysone, 20-hydroxyecdysone (20E) and 24(28)-dehydromakisterone A (dhMaA) were not detected under the analytical conditions used (Supplementary Fig. 6).

## Discussion

The previous misinterpretation of *Monascus* sp. as the fungus eaten by *S. depilis* larvae probably occurred due to inoculation of cerumen fragments during the culturing process coupled to the prior use of media that did not support the growth of osmophilic microorganisms^4^. Despite the presence of *M. ruber* SDCP1 in brood cells cerumen, this fungus was not found in the microbial mass eaten by *S. depilis* larvae. However, the repeated isolation of *Monascus* strains from *S. depilis* cerumen^4^ and other stingless bee colonies^17^ suggests an ecological significance of fungi from this genus to Meliponini bees. Interestingly, *S. depilis*-associated *M. ruber* is highly similar to *M. ruber* strains isolated from the stingless bee *Melipona scutellaris* nests collected in the Northeast of Brazil^17^ (Fig. 2, Table 1). Further investigations are necessary to determine the roles played by *Monascus* spp. inside colonies of stingless bees.

*S. depilis*-associated *Zygosaccharomyces* sp. requires high carbohydrate content and an acidic pH to develop, conditions found in *S. depilis* sugar-rich brood cells^18^. This fungus displays pseudomycelium formation, unprecedented for the genus^16^, flotation and agar invasion (Fig. 3). In brood cells, pseudomycelium and flotation are relevant characteristics of *Zygosaccharomyces* sp. that make it more available to the larvae. The agar invasion is correlated with the necessity of the fungus to pass the cerumen barrier, and thus, finding the lumen of brood cells. Ascospore formation suggests a possible form of dissemination inside *S. depilis* colonies and between colonies during swarming. Apparently, sporulation does not occur during the aerial phase inside brood cells, probably due to the high availability of nutrients. All these growth characteristics show *Zygosaccharomyces* sp. adaptation to survive and proliferates inside *S. depilis* brood cells, further supporting the specificity of this symbiosis.

The requirement for ergosterol provided by *Zygosaccharomyces* sp. (Fig. 4e) reveals the intimate dependence of *S. depilis* on this fungal symbiont. Considering the necessity of steroid sources, *S. depilis*-*Zygosaccharomyces* sp. symbiosis may have originally evolved as a nutritional/hormonal supply. Insects biosynthesize fatty acids, but they lack the genes required to produce key enzymes for the final stages of steroids biosynthesis^19^. They must consume dietary sterols to survive and this nutritional dependence has driven insects to establish intimate relationships with steroid-producing organisms^20^.

Dietary sterols can be used by insects to produce other steroid-like structures in endogenous metabolizing pathways. One of the most widespread sterol-derived hormone is the ecdysone hydroxylated metabolite 20E (Fig. 4f), responsible for larval maturation and morphogenesis through tissue-specific transcriptional cascade activation^21^. The production of the C_27_ ecdysteroid 20E starts with cholesterol (Fig. 4f), which is metabolized by enzymes present in prothoracic glands^22^. Besides the participation of ecdysteroids in the development of insects, sesquiterpenoids known as juvenile hormones (JHs) are also required. JHs are biosynthesized by insects in the corpora allata gland, and down-regulate metamorphosis. The balance between ecdysteroids and JHs coordinates insect development and maturation^14^.

Certain insects catalyze 24-dealkylation in phytosterols^23^ and fungal steroids^24,25^ to generate cholesterol-like sterols, which can then be metabolized to produce C_27_ ecdysteroids. However, some species of Hymenoptera, such as the honeybee *Apis mellifera*, cannot catalyze 24-dealkylation in sterols^26^. These bees use the C_28_ ecdysteroid MaA, which is produced from plants steroids, as major molting hormone^27^. The precursor of MaA seems to be campesterol, a 24-alpha-methyl phytosterol^28^. MaA C-24 epimer epi-MaA (Fig. 4f) was found as the major ecdysteroid in the fungus-growing ant *Acromyrmex octospinosus*^29^, which consumes ergosterol, a 24-beta-methyl sterol produced by the fungal cultivar. The 24(28)-dehydroxylated ecdysteroid dhMaA is also a functional hormone found in *Drosophila melanogaster* and could be originated from 24(28)-dehydroergosterol. To sum up, the sterol substrate and the enzymatic apparatus found in different insects directly influence their ecdysteroid profile^15^.

*In vitro* larval development assays, carried out with ergosterol added in *S. depilis* larval food, showed that this sterol stimulates pupation in a similar degree as *Zygosaccharomyces* sp. cells (Fig. 4e). These results indicated that the fungal sterol can join *S. depilis* endogenous pathways to originate ecdysteroids. We analyzed the chemical profile of *S. depilis* pupae using high resolution LC-MS, and the ion corresponding to the 24-methylated ecdysteroid MaA or epi-MaA was detected (Supplementary Fig. 5). Ecdysone, 20E and dhMaA were not detected (Supplementary Fig. 6). These data suggested that *S. depilis* can use C_28_ ecdysteroids like the honeybee *A. mellifera*^27^. Ergosterol-consuming insects produce epi-MaA rather than MaA^15,29^, supporting that epi-MaA is the molting hormone detected in *S. depilis* pupae. The use of ergosterol furnished by *Zygosaccharomyces* sp. represents the fundamental mechanism of a nutritional/hormonal dependence for this intimate bee-fungus symbiosis.

The dependence of *S. depilis* larvae on sterol supply from *Zygosaccharomyces* sp. for morphogenesis highlights the importance of preserving bee-associated microbiota. Studies indicate the negative impacts of antifungal pesticides to bee survival. The synergistic effects of neonicotinoids and fungicides was shown to be lethal for *A. mellifera*^30,31^, *Bombus terrestris* and *Osmia bicornis*^31^. Frequently, neonicotinoids and fungicides are detected in apicultural products^32–34^, and these compounds could contribute to the decline of bees. Agricultural azoles, such as propiconazole and tebuconazole, act disrupting ergosterol production by blocking the CYP51 sterol 14-alpha-demethylase^35^. The depletion of ergosterol biosynthesis decreases fungus viability and could negatively impact *S. depilis* larval development. More efforts should be done to test antifungal pesticides safety, mainly to wild bees, which are commonly neglected in these studies.

The interactions between yeasts and insects are ancient, but still poorly understood. Fungus-growing ants of the genus *Cyphomyrmex* cultivate basidiomycetous yeast as a food source^36^. A Saccharomycete engages in a symbiotic relationship with the non-social beetle *Doubledaya bucculenta* and seems to be important to the larval development^37^. *Drosophila melanogaster* is a vector of a yeast-community in banana fruits, where their larvae develop^38^. Fungi in the genus *Zygosaccharomyces* have been previously reported from other bees, including bumblebees^39^, honeybees^40,41^, and stingless bees^41,42^. The *Zygosaccharomyces* strains associated with *S. depilis* from this study form a monophyletic group that is the sister group to a lineage that includes several bee-associated *Zygosaccharomyces* spp. (Fig. 2, Table 1). Taken together, this suggests similar cases of fungus-bee symbioses may exist.

Here, we report the relationship of an osmophilic fungus, which proliferates inside *S. depilis* brood cells, and benefits the stingless bee through the production of a fungal sterol that can be converted into an essential steroid hormone. There is no previous study regarding a bee consuming a fungus as a sterol source to help larval development. The specialized chemical-ecological interaction between *S. depilis* and *Zygosaccharomyces* sp. suggests that the antifungal pesticides used to protect agriculture crops could be having substantial impacts on plant pollinator population through the disruption of the bee-associated fungal community.

## Experimental Procedures

### Insect collection and microbial isolation

The experiments were performed using *S. depilis* colonies maintained in wood hives at University of São Paulo, Faculty of Philosophy, Sciences and Letters of Ribeirão Preto, Ribeirão Preto, São Paulo, Brazil (SISBIO authorization 46555-5, CNPq process 010936/2014-9). For microbial isolation, samples from brood cells (cerumen, larval food and white microorganism growing inside brood cells) were carefully collected and aseptically plated on Potato Dextrose Agar (PDA), International *Streptomyces* Project medium number 2 (ISP-2) with 2% agar and 30 G medium (30 g of glucose, 3 g of malt extract, 3 g of yeast extract and 2 g of agar to 100 mL of deionized water, pH adjusted to pH 4.5 or pH 6.0 with hydrochloric acid 1 N). The 15GF medium (15 g of glucose, 15 g of fructose, 3 g of malt extract, 3 g of yeast extract and 2 g of agar to 100 mL of deionized water, pH adjusted to pH 4.5 or pH 6.0 with hydrochloric acid 1 N) was also used for osmophilic microorganism growth. The plates were incubated at 30 °C until microbial growth. *Zygosaccharomyces* sp. lineages were isolated from four different *S. depilis* colonies.

### Nucleic acid extraction for amplification and sequencing

The samples of fungus from 20 brood cells of *S. depilis* were collected, pooled and frozen in liquid nitrogen in a 0.5 mL tube. To this sample, 100 μL of lysis buffer (200 mM Tris-HCl pH 7.5, 250 mM NaCl, 1 mM EDTA and 1% SDS, w/v) was added and mixed thoroughly. The sample was subjected to six alternate cycles of freezing in liquid nitrogen and incubation at 90 °C for 1 min, followed by freezing and a final incubation at 90 °C for 10 min. The tube was centrifuged at 11000 g for 3 min and the supernatant collected carefully. The supernatant was passed through a DNA purification column (QIAquick, Qiagen) according the manufacturer instructions. The purified DNA was eluted in 50 μL of 1 mM Tris-HCl pH 8.5 prewarmed to 55 °C. DNA extractions from isolated microorganisms were performed using FastDNA SPIN Kit for soil (MP), according the manufacturer instructions.

Isolated strains were identified by 18 S rRNA sequencing using the primers NS1 (5′-GTAGTCATATGCTTGTCTC-3′) and NS4 (5′-CTTCCGTCAATTCCTTTAAG-3′)^43^, and D1/D2 26 S rRNA sequencing using the primers NL1 (5′-GCATATCAATAAGCGGAGGAAAAG-3′) and NL4 (5′-GGTCCGTGTTTCAAGACGG-3′). The 18 S sequences were also used to design specific primers for these fungi (Supplementary Table 1). Additionally, for *M. ruber* SDCP1 identification, it was used a taxonomic methodology^44^.

### Phylogenetic analyses

For phylogenetic analyses, the evolutionary history was inferred by using the Maximum Likelihood method based on the Tamura-Nei model^45^. The tree with the highest log likelihood was used. Bootstrap values (1000 bootstraps) are shown as a percentage. Initial tree(s) for the heuristic search were obtained automatically by applying Neighbor-Join and BioNJ algorithms to a matrix of pairwise distances estimated using the Maximum Composite Likelihood (MCL) approach, and then selecting the topology with superior log likelihood value. The tree is drawn to scale, with branch lengths measured in the number of substitutions per site. Evolutionary analyses were conducted in MEGA7^46^.

### Extraction and analyzes of *Zygosaccharomyces* sp. sterols

*Zygosaccharomyces* sp. SDBC30G1 was cultivated in 30G agar medium (pH 6.0), for 15 days at 29 °C. Then, the cells were harvested and washed twice with sterile deionized water, and a cell suspension of OD_600_ 32 was prepared. From this suspension, 250 µL were taken and extracted with 750 µL of chloroform:methanol (2:1) solution. The mixture was vortexed for 2 min, incubated in ice for 30 min and centrifuged for 3 min at 13000 rpm. The organic phase was analyzed at the Chromatography and Mass Spectrometry Center (CEMMO-FCFRP-USP) using a Shimadzu QP-2010 GC-MS. The column applied was the Rtx-5MS (RESTEK) (30 m × 0.25 mm × 0.25 µm), injection temperature of 260 °C, interface temperature of 300 °C, ionization source at 250 °C, splitless mode. The gradient was 60 °C for 4 min, increasing 10 °C per min until 300 °C and remaining at 300 °C for 20 min.

### Larvae *in vitro* culturing with *Zygosaccharomyces* sp

Larval food from brood cells containing eggs was aseptically collected, sterilized by freeze-thawing (−80 °C for 20 min followed by 60 °C for 3 min), and 33 μL of this larval food were transferred to individual wells of a 96-well plate. Then, *S. depilis* eggs were carefully transferred to each well. The plates were incubated at 29 °C for 3–4 days, until the eggs hatched. The inocula of *Zygosaccharomyces* sp. SDBC30G1 were prepared using 30G broth (pH 6.0) from 10–15 day-old cultures in 30 G agar medium (pH 6.0) at 29 °C. Several inocula were tested, and the best results were obtained from inocula with OD_600_ 45, which 1 μL resulted in a final inoculum of approximately 1 × 10^6^ CFU.mL^−1^ in each well containing larva. The inoculum prepared from *Zygosaccharomyces* sp. cells collected directly from brood cells presented a higher cell viability and grew faster than the inoculum prepared from *Zygosaccharomyces* sp. maintained in laboratory. So, a smaller inoculum from the brood cells resident fungus (OD_600_ 38) was used, and 1 μL from this inoculum also gave approximately 1 × 10^6^ CFU.mL^−1^ in each well containing larva. For the treatment without fungus (negative control), 1 μL of 30 G broth (pH 6.0) was transferred to the wells containing larvae. The cultures were monitored for 40–45 days, until the complete larval development. The experiments were performed three times, using eggs and resident fungus from different colonies of *S. depilis*.

### Larvae *in vitro* culturing with ergosterol

The procedure described for larvae culturing with *Zygosaccharomyces* sp. was also performed for larval *in vitro* assays with ergosterol (Sigma-Aldrich). The sterol was dissolved in ethyl acetate and transferred to the larval food to give final concentrations of 2.5 and 0.25 µM (maximum of 2% ethyl acetate in the larval food). As positive control, larvae were reared with cells of *Zygosaccharomyces* sp. collected directly from *S. depilis* brood cells (OD_600_ 38); and as negative control, larvae were reared without fungus or sterol. To eliminate the possibility of ethyl acetate interference, the positive and negative controls also received 2% of the solvent.

### *Zygosaccharomyces* sp. lipid droplets staining

*Zygosaccharomyces* sp. LDs staining was performed according Hoiczyk and collaborators^47^. Fixed cells in a glass slide were stained for 30 min with 50 μL of Nile Red (Sigma-Aldrich) in ethanol (10 μg.mL^−1^), washed briefly with water and observed on confocal microscope Leica SP5 at the Multiuser Laboratory of Confocal Microscopy, University of São Paulo (LMMC, FAPESP 2004/08868-0). Cells were taken from a *Zygosaccharomyces* sp. SDBC30G1 culture in 15GF broth (pH 4.5, seven days old culture at 30 °C, static condition, initial inoculum OD_600_ 0.1), and directly from *S. depilis* brood cells.

### Extraction and metabolic profiling of *S. depilis* pupae

*S. depilis* pupae from three different colonies were collected directly from brood cells and frozen using liquid nitrogen. The material from each colony was treated separately. The pupae were triturated and a sample of 100 mg was sonicated with 1 mL of methanol in a 2 mL centrifuge tube for 10 min, and extracted for 60 min in room temperature. Solvent was dried, the crude extract was dissolved with 1 mL of methanol:water (1:9 v/v) and cleaned by solid-phase extraction (1 mL LC-C_18_ SPE Tubes, 100 mg, Supelclean^TM^). Analyses were performed using a Shimadzu UFLC coupled to a Phenomenex C_18_(2) Luna (5 µm; 100 A; 250 × 4.6 mm) column and with the micrOTOF Q II Mass Spectrometer (Bruker Daltonics, EUA). Samples (20 µL) were analyzed using a gradient of acetonitrile and water, both containing 0.1% of formic acid, as follows: 0–5 min isocratic of 10% of acetonitrile, gradient from 10% of acetonitrile in 5 min to 100% of acetonitrile in 60 min, isocratic of 100% of acetonitrile until 70 min, gradient to 10% of acetonitrile from 70 to 75 min, and isocratic of 10% of acetonitrile until 85 min; flow of 1 mL.min^−1^. For the HR-ESI-MS analysis, plate voltage was 500 V, capillary voltage was 3.5 kV, dry temperature was 220 °C, and dry gas (N_2_) flow was 10 mL.min^−1^.

### Statistical analyses

We performed Cochran-Mantel-Haenszel test using the XLSTAT software, version 2016. All tests were carried out in three independent replicates, each replicate an *n* equal to 16, resulting in a total of 48 larvae for each treatment. The calculated *P* values were reported in the results and figure legends.

### Data availability

The 18 S sequences from *Zygosaccharomyces* sp. SDBC30G1, fungal sample collected from *S. depilis* brood cells and *M. ruber* SDCP1 were deposited in the GenBank nucleotide database with accession numbers KX999554, KX999556 and KX999557, respectively. The D1/D1 26 S sequences from *S. depilis*-associated *Zygosaccharomyces* sp. strains and *M. ruber* SDCP1 were also deposited in the GenBank nucleotide database and the accession numbers are described in the Table 1.

## Electronic supplementary material

Supplementary Information

Movie S1

## References

[1] Peters RS (2017). Evolutionary History of the Hymenoptera. Curr. Biol..

[2] Cardinal S, Danforth BN (2013). Bees diversified in the age of eudicots. Proc. R. Soc. B.

[3] DeWeerdt S (2015). The beeline. Nature.

[4] Menezes C (2015). A Brazilian social bee must cultivate fungus to survive. Curr. Biol..

[5] Flechtmann, C. H. W. & Camargo, C. A. Acari associated with stingless bees (Meliponidae, Hymenoptera) from Brazil. *Proc. 4th Int. Congr. Acarol*., 315–319 (1974).

[6] Menezes, C., Vollet-Neto, A., Contrera, F. A. F. L., Venturieri, G. C. & Imperatriz-Fonseca, V. L. in *Pot-Honey: A legacy**of stingless bees* (eds Vit, P., Pedro, S. R. M. & Roubik, D.) 153–171 (Springer, 2013).

[7] Van Arnam, E. B., Currie, C. R. & Clardy, J. Defense contracts: molecular protection in insect-microbe symbioses. *Chem. Soc. Rev*., 10.1039/c7cs00340d (2018). 10.1039/c7cs00340d 28745342

[8] Oh DC, Poulsen M, Currie CR, Clardy J (2009). Dentigerumycin: a bacterial mediator of an ant-fungus symbiosis. Nat. Chem. Biol..

[9] Weber NA (1966). Fungus-Growing Ants. Science.

[10] De Fine Licht HH, Boomsma JJ, Tunlid A (2014). Symbiotic adaptations in the fungal cultivar of leaf-cutting ants. Nat. Commun..

[11] Currie CR, Scott JA, Summerbell RC, Malloch D (1999). Fungus-growing ants use antibiotic-producing bacteria to control garden parasites. Nature.

[12] Currie CR, Poulsen M, Mendenhall J, Boomsma JJ, Billen J (2006). Coevolved crypts and exocrine glands support mutualistic bacteria in fungus-growing ants. Science.

[13] Dillon RJ, Vennard CT, Charnley AK (2000). Exploitation of gut bacteria in the locust. Nature.

[14] Dubrovsky EB (2005). Hormonal cross talk in insect development. Trends Endocrinol. Metab..

[15] Lavrynenko O (2015). The ecdysteroidome of *Drosophila*: influence of diet and development. Development..

[16] James, S. A. & Stratford, M. In *The Yeasts, a**Taxonomic Study* (eds. Kurtzman, C. P., Fell, J.W. & Boekhout, T.) 937–947 (Elsevier 2011).

[17] Barbosa RN (2017). Phylogenetic analysis of *Monascus* and new species from honey, pollen and nests of stingless bees. Stud. Mycol..

[18] Hartfelder K, Engels W (1989). The composition of larval food in stingless bees: evaluating nutritional balance by chemosystematic methods. Insectes Soc..

[19] Clark AJ, Bloch K (1959). The absence of sterol synthesis in insects. J. Biol. Chem..

[20] Behmer, S. T. & Nes, W. D. In *Advances**in**insect**physiology* (ed. Simpson, S. J.) 1–72 (Elsevier 2003).

[21] Li T-R, White KP (2003). Tissue-specific gene expression and ecdysone-regulated genomic networks in *Drosophila*. Dev. Cell.

[22] Huang X, Warren JT, Gilbert LI (2008). New players in the regulation of ecdysone biosynthesis. J. Genet. Genomics.

[23] Svoboda JA, Kaplanis JN, Robbins WE, Thompson MJ (1975). Recent developments in insect steroid metabolism. Annu. Rev. Entomol..

[24] Beck SD, Kapadia GG (1957). Insect nutrition and metabolism of sterols. Science.

[25] Clark AJ, Bloch K (1959). Conversion of ergosterol to 22-dehydrocholesterol in *Blattella germanica*. J. Biol. Chem..

[26] Svoboda JA, Herbert EW, Thompson MJ (1983). Definitive evidence for lack of phytosterol dealkylation in honey bees. Experientia.

[27] Feldlaufer MF, Herbert EW, Svoboda JA, Thompson MJ, Lusby WR (1985). Makisterone A: The major ecdysteroid from the pupa of the honey bee. Apis mellifera. Insect Biochem..

[28] Feldlaufer MF, Herbert EW, Svoboda JA, Thompson MJ (1986). Biosynthesis of makisterone A and 20-hydroxyecdysone from labeled sterols by the honey bee. Apis mellifera. Arch. Insect Biochem. Physiol..

[29] Maurer P, Girault J-P, Larchmeque M, Lafont R (1993). 24-Epi-makisterone A (not makisterone A) is the major ecdysteroid in the leaf-cutting ant *Acromyrmex octospinosus* (Reich) (Hymenoptera, Formicidae: Attini). Arch. Insect Biochem. Physiol..

[30] Thompson H, Fryday S, Harkin S, Milner S (2014). Potential impacts of synergism in honeybees (*Apis mellifera*) of exposure to neonicotinoids and sprayed fungicides in crops. Apidologie.

[31] Sgolastra F (2017). Synergistic mortality between a neonicotinoid insecticide and an ergosterol-biosynthesis-inhibiting fungicide in three bee species. Pest. Manag. Sci..

[32] Pettis JS (2013). Crop pollination exposes honey bees to pesticides which alters their susceptibility to the gut pathogen *Nosema ceranae*. Plos One.

[33] Roszko MŁ, Kamińska M, Szymczyk K, JęDrzejczak R (2016). Levels of Selected Persistent Organic Pollutants (PCB, PBDE) and pesticides in honey bee pollen sampled in Poland. Plos One.

[34] Harriet J (2017). Agricultural pesticides and veterinary substances in Uruguayan beeswax. Chemosphere.

[35] Warrilow AG, Parker JE, Kelly DE, Kelly SL (2013). Azole affinity of sterol 14α-demethylase (CYP51) enzymes from *Candida albicans* and *Homo sapiens*. Antimicrob. Agents Chemother..

[36] Mueller UG, Rehner SA, Schultz TR (1998). The evolution of agriculture in ants. Science.

[37] Toki W, Tanahashi M, Togashi K, Fukatsu T (2012). Fungal farming in a non-social beetle. Plos One.

[38] Stamps JA, Yang LH, Morales VM, Boundy-Mills KL (2012). *Drosophila* regulate yeast density and increase yeast community similarity in a natural substrate. Plos One.

[39] Brysch-Herzberg M (2004). Ecology of yeasts in plant-bumblebee mutualism in Central Europe. FEMS Microbiol. Ecol..

[40] Čadež N, Fülöp L, Dlauchy D, Péter G (2015). *Zygosaccharomyces fav*i sp. nov., an obligate osmophilic yeast species from bee bread and honey. Antonie Van Leeuwenhoek.

[41] Saksinchai S (2012). A novel ascosporogenous yeast species, *Zygosaccharomyces siamensis*, and the sugar tolerant yeasts associated with raw honey collected in Thailand. Fungal Divers..

[42] Rosa CA, Lachance M-A (2005). *Zygosaccharomyces machadoi* sp. n., a yeast species isolated from a nest of the stingless bee *Tetragonisca angustula*. Lundiana.

[43] White, T. J., Bruns, T., Lee, S. & Taylor, J. W. in *P*CR *pr*otocol*s:**a guide to methods and applications* (eds. Innis, M. A., Gelfand, D. H., Sninsky, J. J. & White, T. J.) 315–322 (Academic Press, 1990).

[44] Hawksworth DL, Pitt JI (1983). A new taxonomy for *Monascus* species based on cultural and microscopical characters. Aust. J. Bot..

[45] Tamura K, Nei M (1993). Estimation of the number of nucleotide substitutions in the control region of mitochondrial DNA in humans and chimpanzees. Mol. Biol. Evol..

[46] Kumar S, Stecher G, Tamura K (2016). MEGA7: Molecular Evolutionary Genetics Analysis version 7.0 for bigger datasets. Mol. Biol. Evol..

[47] Hoiczyk E (2009). Lipid body formation plays a central role in cell fate determination during developmental differentiation of *Myxococcus xanthus*. Mol. Microbiol..

