# Supplementary material for: Stingless Bee Larvae Require Fungal Steroid to Pupate
Source: Sci Rep. 2018 Jan 18;8:1122. doi: 10.1038/s41598-018-19583-9 (PMC5773678; doi:10.1038/s41598-018-19583-9)
Supplement: Supplementary file 1 — Supplementary Information [file 41598_2018_19583_MOESM1_ESM.pdf]

Supplementary Information

Stingless Bee Larvae Require Fungal Steroid to Pupate

Camila R. Paludo[1], Cristiano Menezes[2,3], Eduardo A. Silva-Junior[1], Ayrton Vollet-Neto[3], Andres Andrade-Dominguez[4], Gleb Pishchany[4], Lily Khadempour[5,6], Fabio S. do Nascimento[3], Cameron R. Currie[5], Roberto Kolter[4], Jon Clardy[7*], Mônica T. Pupo[1*]

[1]School of Pharmaceutical Sciences of Ribeirão Preto, University of São Paulo, Ribeirão Preto, São Paulo, 14040-903, Brazil;
[2]Brazilian Agricultural Research Corporation, Embrapa Amazônia Oriental, Belém, 66095-100, Brazil;
[3]Department of Biology, FFCLRP, University of São Paulo, Ribeirão Preto, São Paulo, 14040-901, Brazil;
[4]Department of Microbiology and Immunobiology, Harvard Medical School, Boston, Massachusetts, MA 02115, USA;
[5]Department of Bacteriology, University of Wisconsin, Madison, Wisconsin, WI 53706, USA;
[6]Department of Zoology, University of Wisconsin, Madison, Wisconsin, WI 53706, USA;
[7]Department of Biological Chemistry and Molecular Pharmacology, Harvard Medical School, Boston, Massachusetts, MA 02115, USA.

*Correspondence and requests for materials should be addressed to JC (email: jon_clardy@hms.harvard.edu) or MTP (email: mtpupo@fcfrp.usp.br).

Supplementary Figures:

Figure 1. **Characterization of the microorganism eaten by *S. depilis* larvae.** (**a**) Phylogenetic tree of 18S rRNA of the white fungus collected directly from *S. depilis* brood cells (KX999556), *Zygosaccharomyces* sp. SDBC30G1 (KX999554) and *Monascus ruber* SDCP1 (KX999557), using NS1 and NS4 primers. (**b**) Agarose gel electrophoresis of amplicons from 18S region of *Zygosaccharomyces* sp. SDBC30G1 (Z), white fungus collected directly from *S. depilis* brood cells (WF) and *Monascus ruber* SDCP1 (M) using specific primers to *Zygosaccharomyces* sp. SDBC30G1 (18S - Z) and specific primers to *M. ruber* SDCP1 (18S - M).

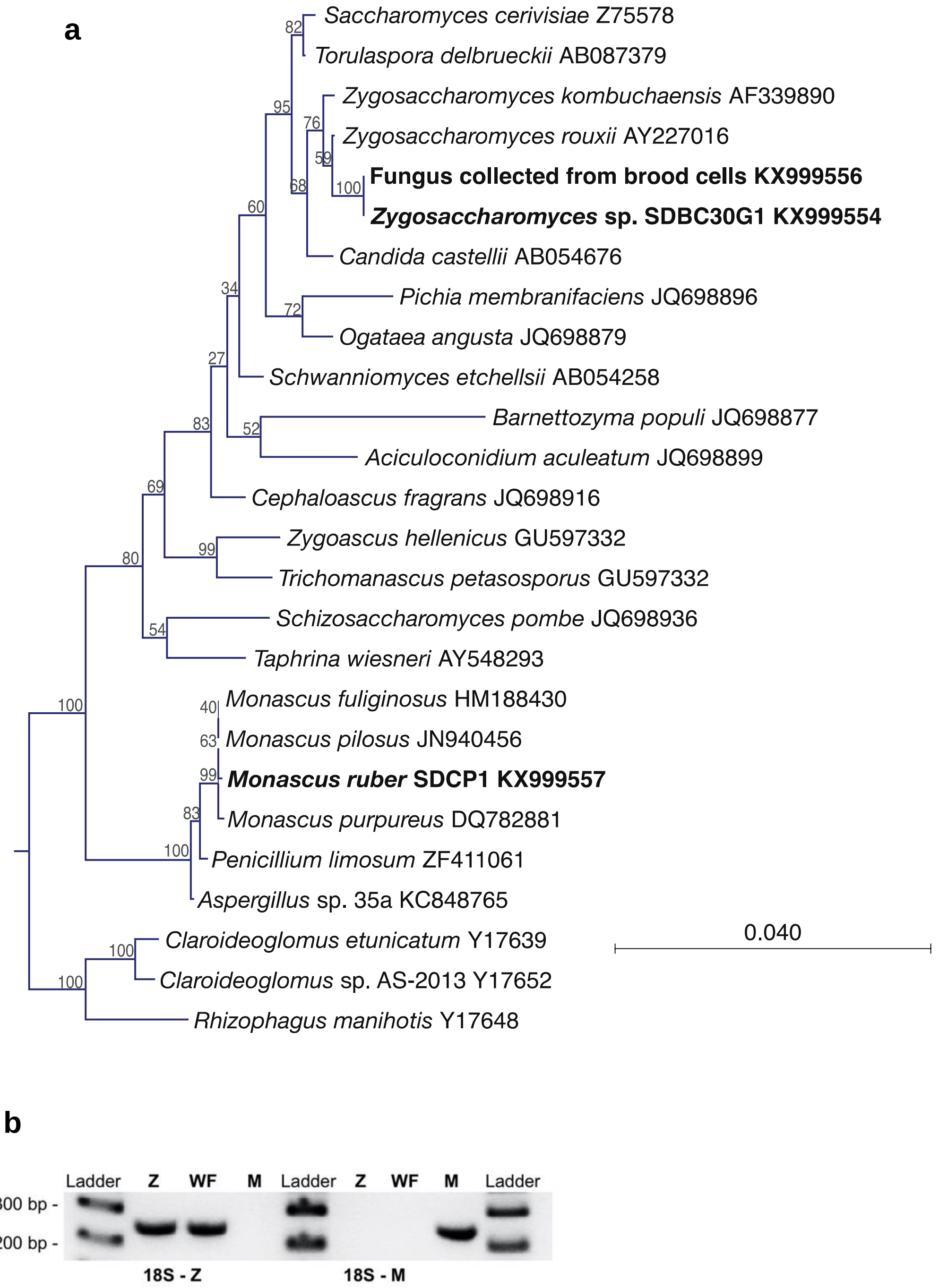

Figure 2. Larval *in vitro* culturing assay using eggs from three different *S. depilis* colonies. (**a**) Larvae reared without fungus inoculation. (**b**) Larvae reared with fungus collected directly from *S. depilis* brood cells. (**c**) Larvae reared with *Zygosaccharomyces* sp. SDBC30G1. # Problem with egg hatching. X Pupation failed.

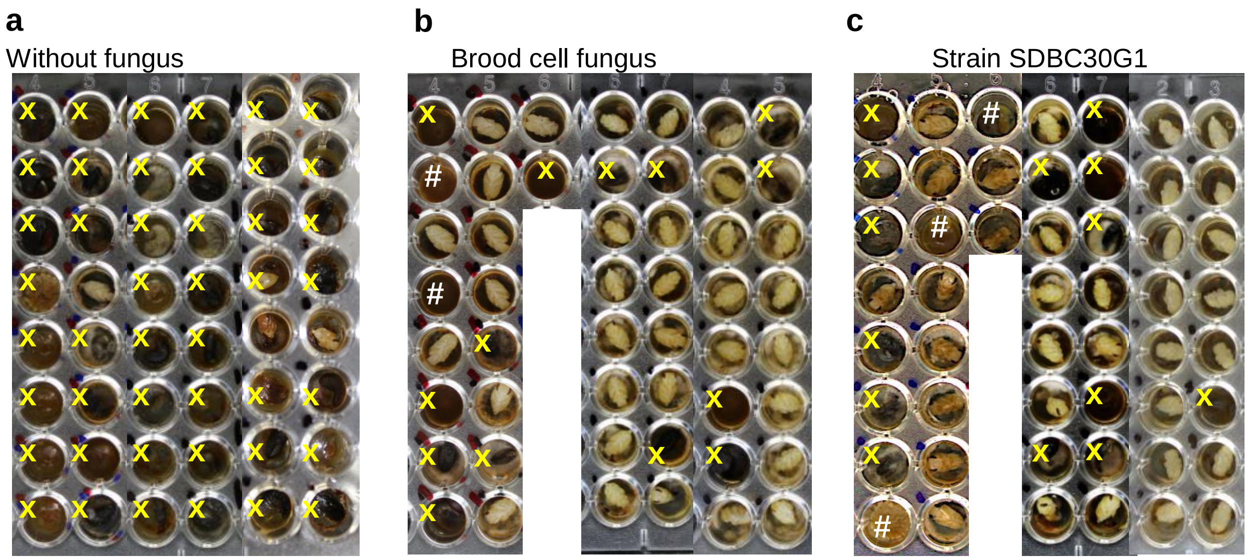

Figure 3. (**a**) GC-MS analysis of *Zygosaccharomyces* sp. SDBC30G1 sterols. (**b**) Identification of ergosterol (30.58 min) using *NIST 11*. Fatty acids detected around 20 min.

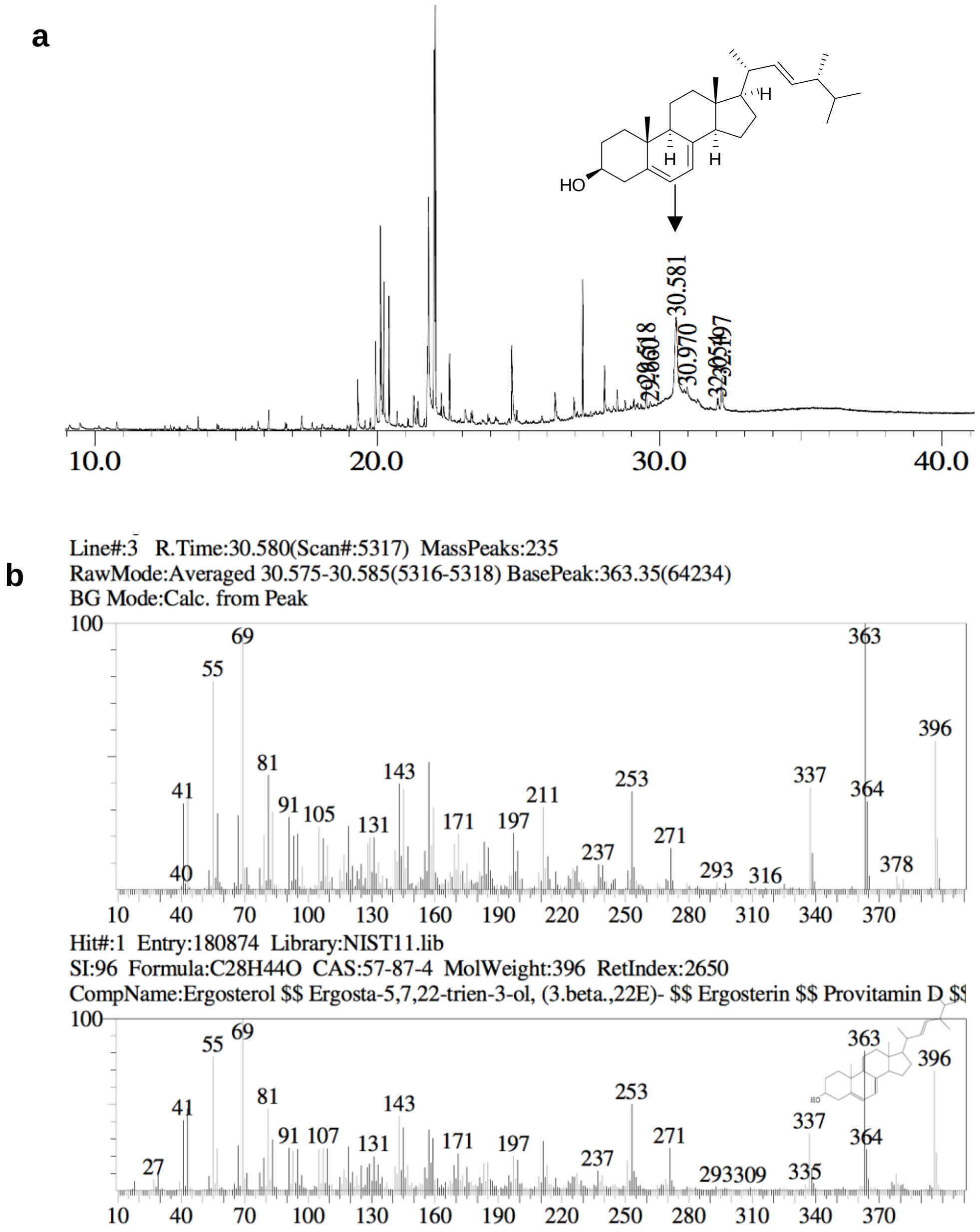

Figure 4. Larval *in vitro* culturing assay using eggs from three different *S. depilis* colonies. (**a**) Larvae reared without fungus or sterol inoculation. (**b**) Larvae reared with fungus collected directly from *S. depilis* brood cells. (**c**) Larvae reared with ergosterol at 2.5 µM in the larval food. X Pupation failed.

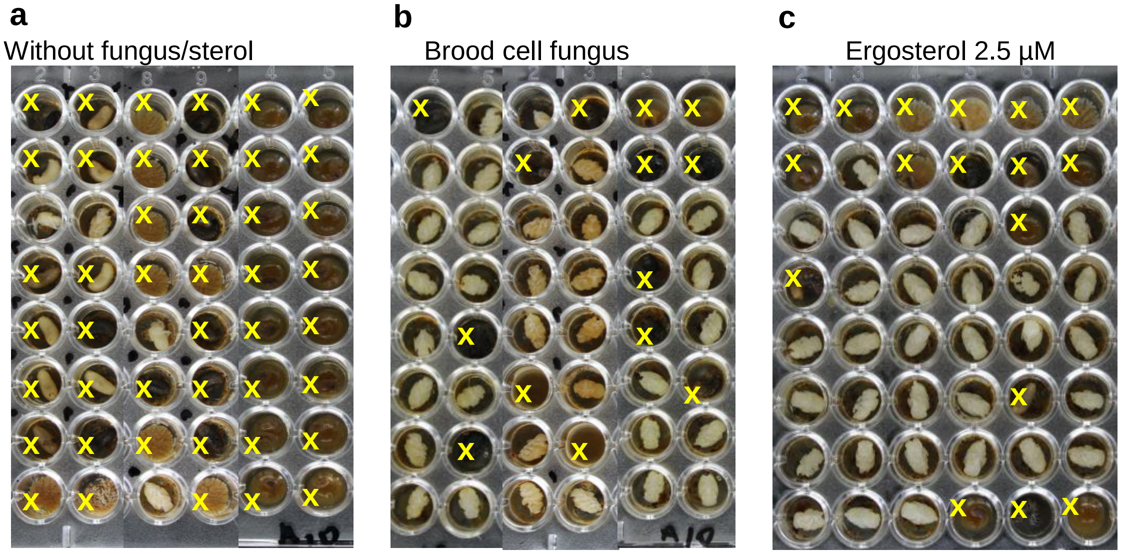

Figure 5. (**a**) Extracted ion chromatogram of *S. depilis* pupae extracts from three different colonies (*m/z* 495.3316). (**b**) HRESIMS of MaA or epi-MaA (*m/z* 495.3314, $C_{28}H_{47}O_7$ $[M+H]^+$, error 0.5 ppm). (**c**) MS/MS of *S. depilis* pupae MaA or epi-MaA.

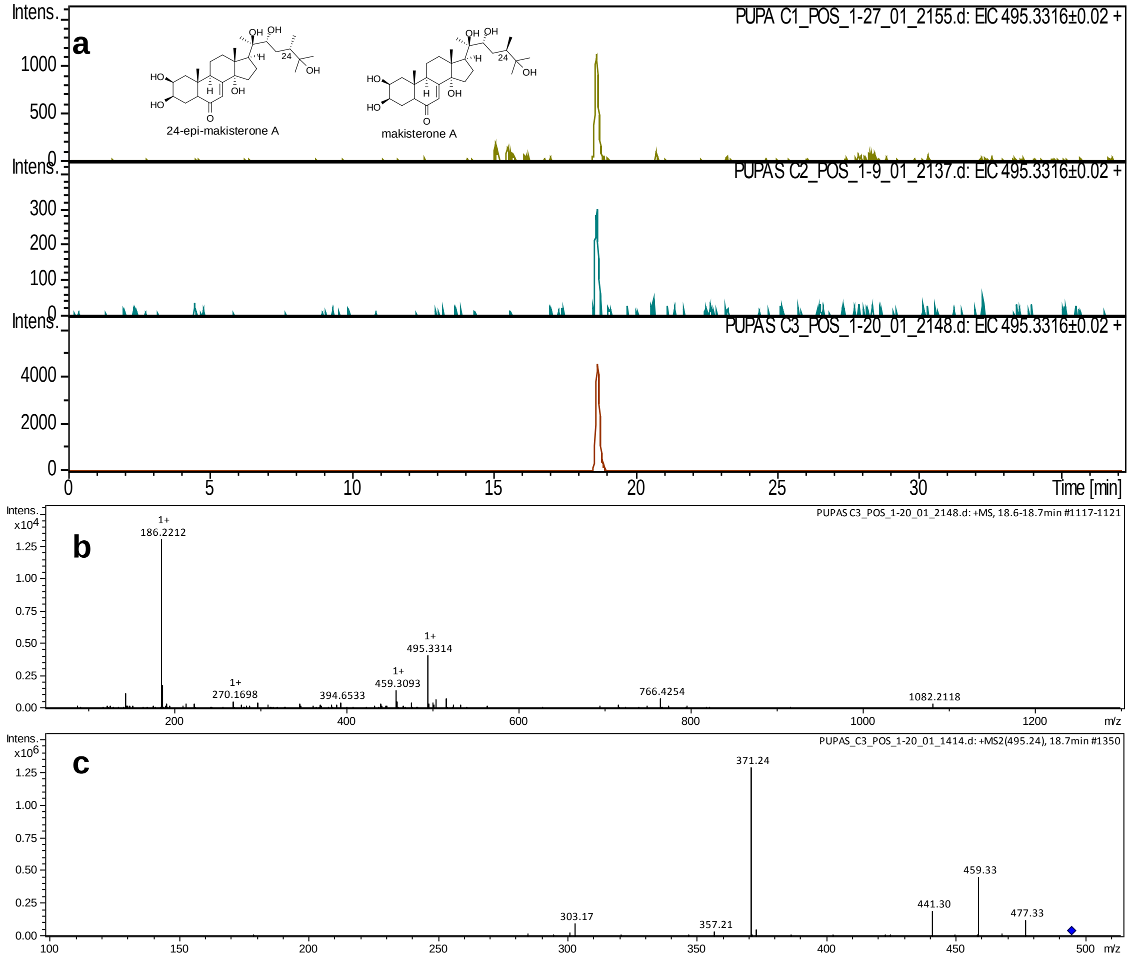

Figure 6. Extracted ion chromatogram from *S. depilis* pupae extract (**a**) *m/z* 495.3316 for MaA or epi-MaA. (**b**) *m/z* 493.3159 for dhMaA. (**c**) *m/z* 481.3159 for 20E. (**d**) *m/z* 465.3210 for ecdysone.

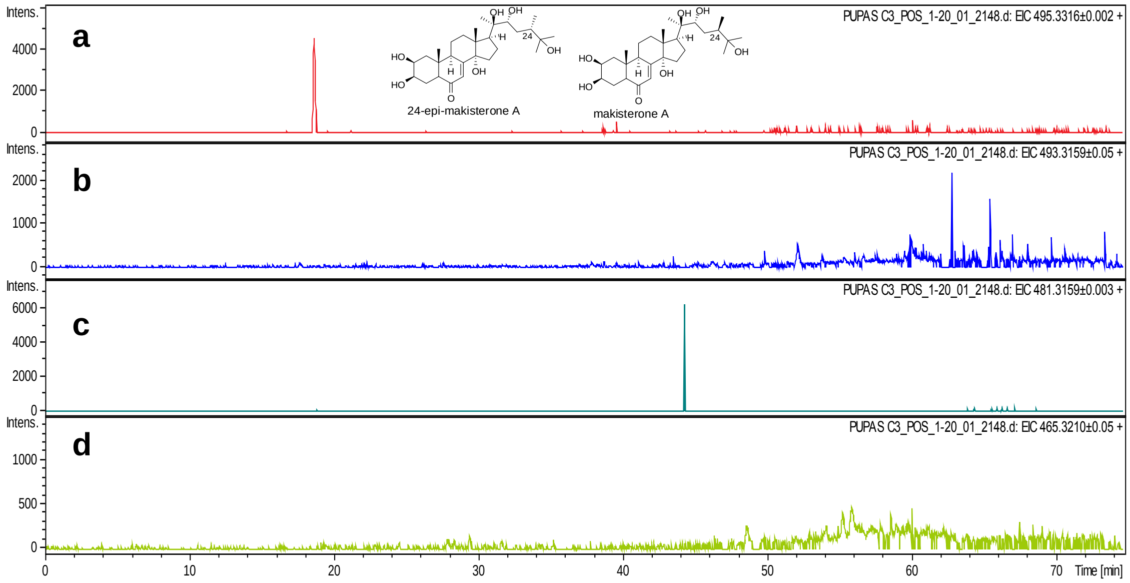

Supplementary table

Table S1. Specific primers for 18S region of *Zygosaccharomyces* sp. SDBC30G1 and *M. ruber* SDCP1.

Microorganism	Designed primer
Zygosaccharomyces sp. SDBC30G1	**FMZ 5’-GCATGGAATAATAGAATAGGACG-3’**<br>**RZ 5’-TGGGTCAGTAAATAAACACCAC-3’**
Monascus ruber SDCP1	**FMZ 5’-GCATGGAATAATAGAATAGGACG-3’**<br>**RM 5’-GGTCATCATAGAAACCCGT-3’**

Supplementary movie

Movie S1. Time lapse of the flotation phenomenon of *Zygosaccharomyces* sp. SDBC30G1 cells in 15GF broth, pH 4.5.
